# Impact of Obesity Caused by a High-Fat Diet on the Heart’s Redox Balance

**DOI:** 10.3390/antiox14060708

**Published:** 2025-06-11

**Authors:** Yildy Utreras-Mendoza, Isidora Mujica Valenzuela, Luis Montecinos, Paulina Donoso, Gina Sánchez

**Affiliations:** 1 Programa de Fisiología y Biofísica, Instituto de Ciencias Biomédicas, Facultad de Medicina, Universidad de Chile, Avda. Independencia 1027, Santiago 8380172, Chile; yildyutreras@gmail.com (Y.U.-M.); lamontecinos@gmail.com (L.M.); 2 Programa de Fisiopatología, Instituto de Ciencias Biomédicas, Facultad de Medicina, Universidad de Chile, Avda. Independencia 1027, Santiago 8380172, Chile; mujicaisidora@gmail.com

**Keywords:** obesity, high-fat diet, cardiac redox status, glutathione, cardiac hypertrophy

## Abstract

Obesity has been implicated in the induction of oxidative stress, which is thought to contribute to the pathogenesis of various cardiovascular diseases, including cardiac hypertrophy. However, the redox status during the early stages of cardiac hypertrophy remains inadequately characterized. In this study, we administered a high-fat diet (HFD) to C57BL/6N mice for 12 weeks. We investigated the expression of biomarkers associated with hypertrophy and oxidative stress, including lipid peroxidation, protein carbonylation, and the redox couples NADH/NAD^+^, NADPH/NADP^+^, and GSH/GSSG. Additionally, we assessed the expression levels and enzymatic activities of catalase, glutathione peroxidase, glutathione reductase, and superoxide dismutase. Following 12 weeks on a HFD, mice exhibited obesity and a 10% increase in the heart weight/tibia length ratio, together with an upregulation in the mRNA levels of β-myosin heavy chain, brain natriuretic peptide, and regulator of calcineurin 1, isoform 4. There was also a significant increase in NOX4 content in the heart of these animals; however, we observed no rise in protein carbonylation and a decrease in lipid peroxidation products. As for the redox couples, the GSH/GSSG ratio nearly doubled, while the NADH/NAD^+^ and NADPH/NADP^+^ ratios remained stable. All antioxidant enzyme mRNAs examined showed increased expression; however, only glutathione reductase showed higher activity. Our findings suggest that reductive stress is predominant within the cardiac environment of these animals.

## 1. Introduction

Maintaining an optimal equilibrium between oxidants and antioxidants is critical for cellular function. Oxidative stress can elicit adverse cellular responses, while excessive antioxidants or reductive molecules may interfere with signaling pathways modulated by reactive oxygen species (ROS) or cause improper protein functionality.

Obesity is recognized as a significant risk factor for various cardiovascular diseases, including arrhythmias, cardiac hypertrophy, and heart failure [1]. Experimental studies suggest that obesity leads to increased production of ROS, contributing to oxidative stress, which activates several signaling pathways that can ultimately result in cardiac hypertrophy [2,3,4,5].

Feeding rodents a high-fat diet (HFD) is a well-established method to induce obesity and insulin resistance [6,7]. We have shown previously that the expression of NOX4, a hydrogen peroxide-producing enzyme, increases in the hearts of mice fed a high-fat diet [8]. These animals also have an increased occurrence of spontaneous ventricular arrhythmias, which is associated with higher activity of Ryanodine Receptors (RyR2), the calcium release channels of the sarcoplasmic reticulum, in planar bilayers [8]. The increased activity of RyR2 is most likely due to oxidative modifications of the protein, since the reducing thiol reagent dithiothreitol reduces channel activity in bilayers. When HFD-fed mice are given apocynin, a polyphenolic compound with antioxidant and anti-inflammatory properties, cardiac arrhythmias disappear, and the activity of RyR2 isolated from their hearts is not different from the activity of channels isolated from control hearts [8].

In addition to the observed increase in body weight, HFD-fed mice had cardiac hypertrophy as indicated by the enlargement of the heart. This finding initially suggested that cardiac hypertrophy might arise because of oxidative stress mediated by NOX4. However, preliminary investigations revealed no detectable evidence of oxidative stress within the cardiac tissues of these mice. This observation—cardiac hypertrophy occurring in the absence of oxidative stress—challenges the prevailing notion that oxidative stress is a fundamental pathophysiological characteristic associated with cardiac hypertrophy [9].

To address this controversy, we examined the impact of HFD on three crucial redox pairs involved in maintaining normal redox status in the heart. The tripeptide glutathione (GSH) is an essential component of cellular antioxidant defense. In its reduced state, GSH can react directly with free radicals and reactive oxygen species to reduce and detoxify them. GSH is also a cofactor for glutathione peroxidases (GPX), an important antioxidant enzyme that reduces both hydrogen peroxide and organic peroxides preventing the degradation of membrane lipids [10,11,12]. The resultant glutathione disulfide (GSSG) is enzymatically reduced back to GSH by glutathione reductase (GR), which uses NADPH as a cofactor. NADPH is produced by the oxidation of glucose and other enzymes, but in mitochondria, the main site of ROS generation, NADPH is produced by the enzyme Nicotinamide Nucleotide Transhydrogenase (Nnt), which transfers hydride ions from NADH to NADP^+^ [13].

These three molecules, glutathione, NADH, and NADPH, are, therefore, critically linked to each other, and the redox couples NADH/NAD^+^, NADPH/NADP^+^, and GSH/GSSG, together with the enzymes that catalyze their interconversions, play crucial roles in redox homeostasis and its dependence on metabolic pathways.

The aim of his work was to assess the redox status of the heart in mice subjected to a high-fat diet by measuring the key redox couples in cardiac tissue: GSH/GSSH, NADH/NAD^+^, and NADPH/NADP^+^. Furthermore, we investigated the expression and activity of key antioxidant enzymes, including superoxide dismutase, catalase, glutathione peroxidase, and glutathione reductase. Understanding the impact of a high-fat diet on the response of the cardiac antioxidant system is important for exploring therapeutic strategies for obesity-related cardiovascular diseases.

## 2. Materials and Methods

### 2.1. Ethical Approval

All procedures in this work were performed in accordance with the Guide for the Care and Use of Laboratory Animals, published by the U.S. National Institutes of Health (NIH Publication, 8th Edition, 2011), and approved by the Institutional Ethics Committee of the School of Medicine, Universidad de Chile (Protocol CBA0819 FMUCH, approved on 2 June 2016).

### 2.2. Animals and Dietary Model

Male C57BL/6N mice (21 days old) were fed with HFD (60% calories from fat, Cat N° D-12492, Research Diets, New Brunswick, NJ, USA) for 12 weeks. In parallel, a control group of mice was fed with a regular diet (10% calories from fat, Champion^®^, Santiago, Chile). During this period, mice were kept at 23 ± 2 °C and a 12:12 h light–dark cycle with free access to food and water. Physical parameters (Table 1) were measured in a larger cohort of animals. Only part of this cohort was used in the experiments shown in this work. In some hearts, the protein available was not enough to measure all the variables shown here. This is the reason why N varies in different determinations.

### 2.3. Obtaining Blood Samples and Preparation of Cardiac Homogenates After 12 Weeks of Control Diet or High-Fat Diet

Mice were subjected to a 6-h fasting period before being anesthetized with isoflurane and euthanized via cervical dislocation. Following thoracotomy, blood samples were obtained using heparinized syringes through cardiac puncture. The hearts were subsequently excised and thoroughly rinsed with Krebs solution to eliminate the remaining blood. The ventricles were rapidly frozen in liquid nitrogen for the preparation of whole homogenates, as detailed previously [14]. The concentration of proteins was quantified utilizing the Bicinchoninic Acid (BCA) assay, employing the Pierce™ BCA Protein Assay Kit (Thermo Scientific™, code number 23227, Rockford, IL, USA).

### 2.4. Determination of Biochemical Parameters in Plasma

The following commercial kits from Human Diagnostic Worldwide, Wiesbaden, Germany, were used: Glucose liquicolor, #10260; Triglycerides liquicolor, #10724; Cholesterol liquicolor #10028; Mouse Ultrasensitive Insulin ELISA, #80-INSMSU-E01, E10, ALPCO, Salem, NH, USA. All procedures were performed according to the manufacturer’s instructions.

### 2.5. Echocardiographic Determinations

Transthoracic M-mode images of the left ventricle were obtained in conscious mice with an echocardiograph equipped with an 8 MHz transducer (ATL 5000 ultrasound machine, Amsterdam, The Netherlands). The internal cavity sizes of the left ventricles at the end of diastole (LVIDd) and systole (LVIDs) were measured, and the fractional shortening of the left ventricle was calculated as ((LVIDd − LVIDs)/LVIDd) × 100. Mice were trained on three consecutive days previous to the echocardiographic examination as described [15].

### 2.6. NOX4 Determination

NOX4 was determined in whole homogenates by Western blot analysis. Proteins were separated in 10% SDS-PAGE gels, transferred to polyvinylidene difluoride (PVDF) membranes, and probed with anti-NOX4 (code 133303, Abcam, Cambridge, UK). GAPDH (code G9545, Sigma Aldrich, St. Louis, MO, USA) was used for normalization.

### 2.7. Lipid Peroxidation

To determine lipid oxidation, thiobarbituric acid reactive species (TBARS) were quantified in cardiac tissue homogenate with the Lipid Peroxidation Kit (ab233471, ABCAM, Waltham, MA, USA) according to the manufacturer’s instructions.

### 2.8. Protein Carbonylation

Cardiac homogenates were derivatized by incubation with 2,4 dinitro phenyl hydrazine (DNPH) using the Protein Carbonyl Assay Kit (ab178020, ABCAM, Waltham, MA, USA). The carbonylated proteins were determined in Western blot, after SDS protein electrophoresis, and transferred to PVDF membranes, with the antibody provided by the manufacturer.

### 2.9. Glutathione Quantification

Glutathione quantification was performed according to Griffith [16], and the ratio GSH/GSSG was calculated. This method is based on the kinetic determination of the reaction of glutathione with glutathione reductase and NADPH in the presence of 5,5′-dithiobis-2-nitrobenzoic acid (DTNB). The absorbance of a yellow DTNB-reduced product was measured at 410 nm in a plate reader Synergy 2 (Biotek, Winooski, VT, USA). GSSG was detected after reaction with 2-vinylpyridine (1 h in the dark) to alkylate GSH and determined as above. Reduced GSH was calculated as the difference between total glutathione minus oxidized glutathione, and the results were expressed per mg protein.

### 2.10. Quantification of Pyridine Nucleotides

We used the NAD/NADH Assay Kit (ab65348, ABCAM, Waltham, MA, USA) and the NADP/NADPH Assay Kit (ab65349, ABCAM, Waltham, MA, USA), following the instructions provided by the manufacturer.

### 2.11. RNA Isolation and qRT-PCR

Total RNA was isolated from mouse ventricles using PureZOLTM (Bio-Rad, Hercules, CA, USA) according to the manufacturer’s instructions. RNA from each sample was used for real time (RT) using the iScriptTM cDNA synthesis kit (Bio-Rad). The cDNA was used for quantitative polymerase chain reaction (PCR) analysis in an amplification system (MX3000P, Stratagene, La Jolla, CA, USA) using Brilliant III Ultra-Fast SYBR^®^ Green QPCR Master Mix (Agilent, Santa Clara, CA, USA). Values were normalized to the expression of the constitutive 18s rRNA (Eukaryotic 18S rRNA Endogenous Control, 4319413E, Thermo Fischer Scientific, Rockford, IL, USA). The 2^−ΔΔCt^ method was used to calculate relative transcript abundances. Primers used are shown in Table 1**.**

### 2.12. Enzymatic Activities of Antioxidant Enzymes

To determine the activity of the antioxidant enzymes, we used the following kits: Catalase (ABCAM, ab118184, Waltham, MA, USA), Superoxide Dismutase (Cayman Chemical, 706002, Ann Arbor, MI, USA), Glutathione Peroxidase (ABCAM, ab102530, Waltham, MA, USA), and Glutathione Reductase (ABCAM, ab83461, Waltham, MA, USA).

### 2.13. Statistical Analysis

Data are expressed as mean ± SD (Table) or SEM (Graphics). The Shapiro–Wilk test was used to evaluate normal distribution to all data sets. The data was analyzed by Student’s *t*-test. All statistical analysis was performed using GraphPad Prism version 5 for Windows, GraphPad software, La Jolla, CA, USA. Differences were considered significant at *p* < 0.05.

## 3. Results

### 3.1. Effect of HFD on Mice’s Physical, Biochemical, and Echocardiographic Values

Mice fed with a high-fat diet (HFD) for 12 weeks showed a significant increase in body weight and heart weight compared to the control group (Table 2). They also present hyperglycemia, hypertriglyceridemia, hypercholesterolemia, and hyperinsulinemia. In HFD-fed mice, the heart weight/tibia length ratio was 10% higher than in the control group. Since the tibia length was the same in both groups, this ratio indicates cardiac hypertrophy in the HFD-fed animals (Table 2). However, echocardiograms performed in these mice showed no differences in cardiac dimensions between mice fed a control or a high-fat diet. The ventricular internal dimensions in systole and diastole and the calculated shortening fraction indicate a normal systolic function in obese mice (Table 3).

### 3.2. HFD Induces the Appearance of Markers Indicative of Pathological Hypertrophy

**Figure 1:**
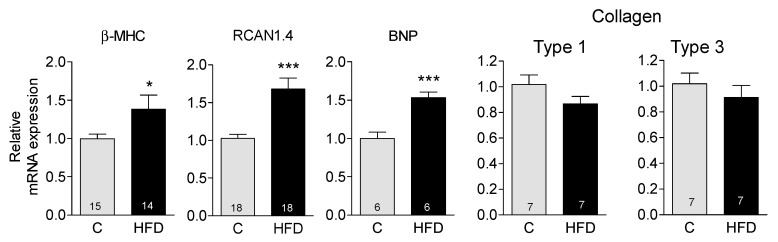
Effect of HFD on the mRNA expression of cardiac hypertrophy markers: mRNA expression determined by RT-qPCR in cardiac homogenates of β-MHC (β-myosin heavy chain); RCAN 1.4 (Regulator of Calcineurin 1 isoform 4); BNP (Brain Natriuretic Peptide); Collagen type 1 and Collagen type 3. Data, expressed relative to the 18s rRNA, is shown as mean ± SEM. The number of experiments is shown in each bar. * *p* < 0.05; *** *p* < 0.001.

The cardiac hypertrophy observed in HFD-fed mice may represent a physiological adaptation to their increased size; however, it may also indicate the onset of pathological hypertrophy. To differentiate between these two conditions, we measured the mRNA levels of three well-known markers of pathological hypertrophy—myosin heavy chain β-isoform (β-MHC), regulator of calcineurin 1.4 (RCAN 1.4), and brain natriuretic peptide (BNP)—in heart homogenates using RT-qPCR. Our analysis revealed that all three markers significantly increased in cardiac tissue following 12 weeks of HFD (Figure 1). In contrast, the mRNAs for collagen types 1 and 3 did not differ from control levels, indicating the absence of fibrosis in these animals and that the hypertrophy is still in an early stage (Figure 1).

### 3.3. Evaluation of Oxidative Stress in Cardiac Tissue

**Figure 2:**
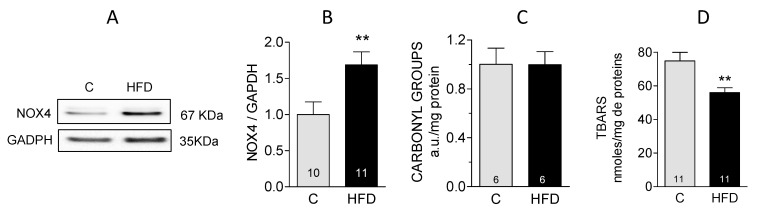
Effect of HFD on NOX4 content and oxidative stress markers: (**A**): Representative Western blots of NOX4; (**B**): quantification of Western blot like those shown in (**A**); (**C**): carbonyl groups in proteins measured as the incorporation of dinitrophenylhydrazine; (**D**): thiobarbituric acid reactive substances (TBARS) content. In each graph, bars represent the mean ± SEM of the number of experiments shown in the bars. ** *p* < 0.01.

Our previous findings indicated that the expression of NOX4 in the cardiac tissue of mice subjected to a HFD increased by 36% after eight weeks [8]. By twelve weeks, the increase in NOX4 reached 75% (Figure 2A,B), suggesting that oxidative stress increases when mice receive the HFD for a longer period. To assess the degree of oxidative damage to proteins and lipids in the hearts of HFD mice, we quantified total carbonylated proteins and thiobarbituric acid reactive substances (TBARS). Contrary to what we expected, the results revealed no significant alteration in the levels of carbonylated proteins as determined by the incorporation of dinitrophenylhydrazine (DNP) (Figure 2C). Moreover, we observed a significant decrease in TBARS levels in HFD mice compared to control subjects (Figure 2D). These findings imply that, despite the elevated NOX4 expression, there is no evidence of oxidative stress in mice hearts after 12 weeks of HFD.

### 3.4. Effect of HFD on Redox Buffers in Cardiac Tissue

**Figure 3:**
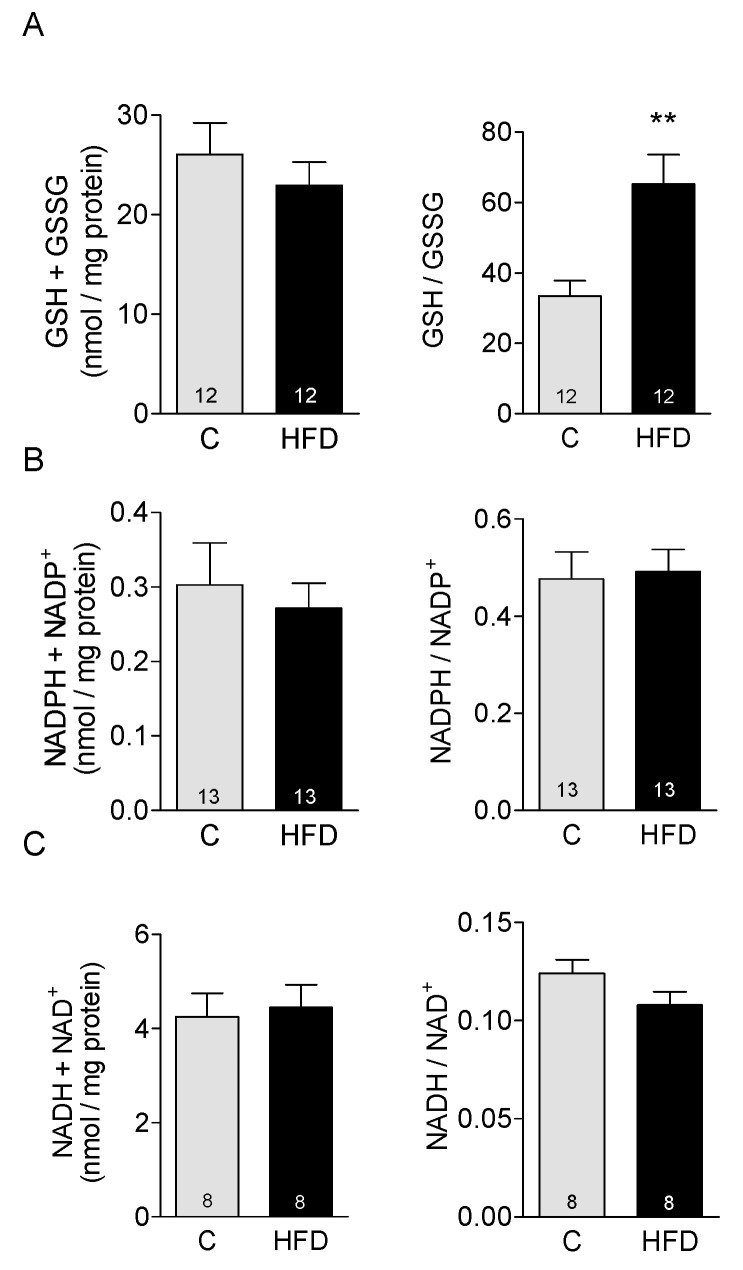
Effect of HFD on redox buffers in cardiac tissue: Quantification of the total concentration (left panel), and the reduced/oxidized ratio (right panel) of (**A**): Glutathione, (**B**): NADPH, and (**C**): NADH, measured in cardiac homogenates. The number of experiments is shown in the bars ** *p* < 0.01 vs. control.

To evaluate the effect of HFD on the main redox buffers in cardiac tissue, we measured the total concentration of glutathione, pyridine nucleotides, and the ratio of reduced/oxidized redox pairs. There were no changes in the total concentration of any of these redox buffers in HFD compared to control mice (Figure 3A–C), but there was a significant increase in GSH/GSSG ratio (Figure 3, right panel), denoting a more reduced environment in the cardiac tissue of HFD-fed mice. For pyridine nucleotides, there were no changes in NADPH/NADP^+^ ratio (Figure 3B right panel), or in NADH/NAD^+^ ratio in HFD-fed mice (Figure 3C right panel).

### 3.5. Effect of HFD on Antioxidant Enzymes in Cardiac Tissue

**Figure 4:**
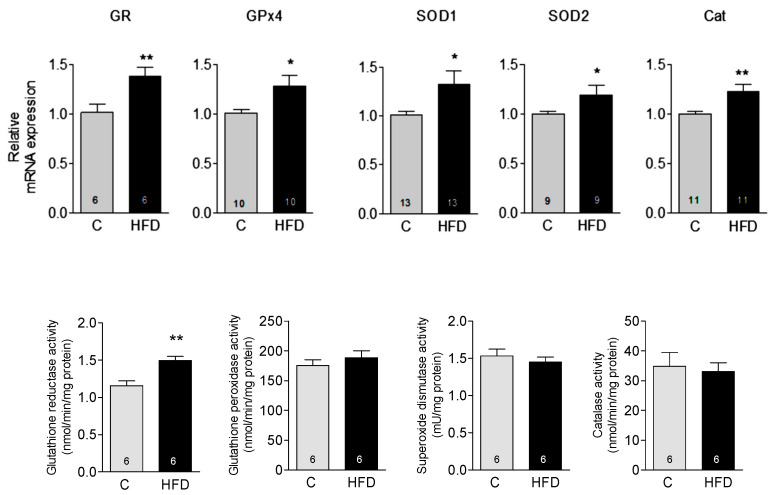
Effect of HFD on antioxidant enzymes in cardiac tissue: Gene expression of glutathione reductase (GR), glutathione peroxidase 4 (GPX4), SOD1 (Superoxide dismutase 1), SOD2 (Superoxide dismutase 2) and catalase was determined in cardiac homogenates by RT-qPCR. Data, expressed relative to the 18s rRNA, are shown as mean ± SEM. Enzymatic activity is shown in the lower panel. The number of experiments is shown in each bar. * *p* < 0.05; ** *p* < 0.01 vs. control.

An important aspect of redox status in cells is the expression and activity of antioxidant enzymes. We quantified the gene expression and the activity of some relevant antioxidant enzymes in heart tissue. We found a small but significant increase in the mRNA expression of catalase, SOD 1 (cytoplasmic) and SOD 2 (mitochondrial), glutathione peroxidase 4, and glutathione reductase in heart homogenates of HFD mice, determined by RT-qPCR (Figure 4, upper panel). However, when evaluating the enzymatic activities, only the activity of glutathione reductase was significantly increased, indicating that the high-fat diet effectively enhanced the activity of this specific enzyme (Figure 4 lower panel).

## 4. Discussion

The increased workload imposed on the heart by obesity can ultimately lead to cardiac hypertrophy. Various rodent models of diet-induced obesity have been developed to explore the molecular mechanisms behind this condition. However, there is still no complete understanding of this process.

A prominent finding shared by many studies about diet-induced obesity is oxidative stress. Thus, oxidative stress is believed to play a central role in the pathogenesis of cardiac hypertrophy [5,9].

In this work, mice fed a high-fat diet for 12 weeks presented all the expected metabolic alterations with this type of diet, i.e., increase in body weight, hyperglycemia, hyperlipidemia, and hyperinsulinemia, but no oxidative stress damage as assessed by protein carbonylation or lipid peroxidation. The decrease in lipid peroxidation products (TBARS) suggests a lower level of oxidative damage in the hearts of HFD-fed mice compared to controls. Nevertheless, these mice had an increase in cardiac mass and overexpression of the pathological hypertrophy markers β-MHC, RCAN-1.4, and BNP genes. Echocardiographic analysis revealed no significant differences in fractional shortening between the control and the HFD groups, which, together with the absence of fibrosis in the HFD group, suggests that cardiac hypertrophy is still incipient in these mice.

The absence of oxidative stress, together with an increased NOX4 expression in the hearts of mice subjected to a high-fat diet (HFD), suggests that the generation of ROS by NOX4 occurs in discrete areas that are strategically positioned near the intended targets of these ROS. Alternatively, the cellular antioxidant mechanisms could be highly efficient, rapidly neutralizing any ROS produced by NOX4 before they can overcome the cellular antioxidant defenses.

Glutathione is the most abundant cellular thiol and accounts for most of the reducing power in cells. Glutathione constitutes the first line of defense against organic and inorganic peroxides [17]. HFD mice had a significant increase in GSH over its corresponding oxidized species GSSG, indicating that the redox state of cardiac tissue is reduced rather than oxidized. This finding contrasts with reports in the literature that typically associate obesity with oxidative stress. Several factors may account for this discrepancy, including differences in animal models, strains, or dietary protocols that could impact the outcomes observed. In many cases, the redox state has been inferred from other tissues, such as liver, muscle, or adipose tissue, rather than from the heart (reviewed in [18]). C57BL/6J mice is a strain often used in diet-induced obesity protocols. This strain has a deletion in the gene that codifies for the enzyme nicotinamide nucleotide transhydrogenase (Nnt) and therefore has no functional Nnt protein [19]. Mitochondrial Nnt catalyzes the reduction of NADP+ to generate NADPH at expenses of NADH. Mutant mice have lower mitochondrial NADPH, and since this reduced nucleotide is used by glutathione peroxidase to reduce GSSG, C57BL/6J mice also have reduced mitochondrial GSH concentrations and increased rate of hydrogen peroxide generation among other abnormalities [20]. Our experiments were performed on the strain C57BL/6N, which has the normal Nnt enzyme and should have a normal recovery of mitochondrial GSH and better antioxidant defenses.

The excess of reduced glutathione concentration would disturb the normal redox balance in the heart, with harmful consequences to cell function. Increases in the cellular reductive power change the redox state of critical thiols and interfere with the formation of structural disulfide bridges in proteins, altering their activity and producing protein aggregation. Several studies have documented the relationship between reductive stress and the development of cardiac hypertrophy. For instance, a mutation in αB-crystallin, a molecular chaperone found in various mammalian cells, has been associated with hypertrophy and heart failure. Mice expressing a mutated form of αB-crystallin (R120G-αB-crystallin) exhibit increased expression and activity of key antioxidant enzymes, including catalase, GPx-1, and glutathione reductase, along with elevated levels of reduced glutathione, protein aggregation, and hypertrophy [21]. Similar redox responses have been observed with the small heat shock protein Hsp27. In mice that overexpress Hsp27, there is important reductive stress characterized by an elevated GSH/GSSG ratio, increased expression and activity of GPx1, and decreased reactive oxygen species (ROS) levels, which produces cardiac hypertrophy and impaired contractile function [22]. Moreover, transgenic mice expressing a constitutively active form of nuclear erythroid related factor-2 (Nrf2) also develop hypertrophic cardiomyopathy [23]. Nrf2 is a critical regulator of the expression of antioxidant enzymes, and these transgenic models demonstrate that increased antioxidant enzyme levels lead to enhanced GSH content and reductive stress, which cells cannot tolerate.

Changes in the expression of antioxidant enzymes in response to the HFD could also explain the observed increase in reduced glutathione. An increase in mitochondrial catalase expression occurs in mice a short time after starting a high-fat diet [24]. Overexpression of catalase or other antioxidant enzymes can affect redox equilibrium and change the cellular environment towards a more reduced one [25]. In this study, we observed modest increases in the mRNA levels of catalase, superoxide dismutase (both mitochondrial and cytoplasmic), glutathione peroxidase, and glutathione reductase. However, these increases did not lead to enhanced enzymatic activity, except in the case of glutathione reductase, which may account for the rise in reduced glutathione observed in our findings. While elevated mRNA levels can suggest increased functional protein activity, the relationship is complex. The conversion of mRNA into a functional protein is tightly regulated and involves numerous post-translational control mechanisms. Factors such as mRNA stability, processing, and localization within the cell can influence translation. After a protein is synthesized, it must properly fold, undergo post-translational modifications, and be accurately directed within the cell to fulfill its biological role. Especially when there is a rise in reductants in the heart, the production of antioxidant enzymes is likely finely regulated to avoid exacerbating the situation.

The high GSH/GSSG ratio in obese mice found in this work suggests a metabolic effect of fatty acids. In physiological conditions, the cardiac muscle generates approximately 60–80% of the total ATP needed from fatty acid beta-oxidation [26], and even more when the supply of fatty acids is higher [27,28]. The higher availability of fatty acids for β-oxidation in HFD-fed mice would have generated a higher amount of the reducing equivalents NADH and FADH2 [29]. These equivalents are subsequently transferred from NADH to NADPH by the catalytic action of Nnt, and ultimately to GSH in a reaction catalyzed by glutathione reductase, accounting for the observed increase in GSH/GSSG ratio.

Another effect of fatty acids is their ability to diminish mitochondrial ROS production by two mechanisms. First, fatty acids reduce the rate of electron transport within the respiratory chain by inhibiting respiratory complexes, thus decreasing ROS generation [30]. Secondly, fatty acids function as uncouplers, transporting protons across the mitochondrial membrane [30,31]. In their undissociated form, they permeate the inner mitochondrial membrane, release a proton in the mitochondrial matrix, and subsequently return to the cytosol in a deprotonated state linked to mitochondrial uncoupling proteins [31,32]. Cardiac myocytes derived from both control and db/db mice, when exposed to palmitate, exhibit a significant reduction in reactive oxygen species (ROS) levels and a concomitant increase in glutathione (GSH), indicating that fatty acids may either inhibit ROS generation by cardiomyocytes or provide the reducing equivalents necessary for ROS elimination [33]. Therefore, fatty acids can reduce the generation of ROS in the mitochondria and increase the concentration of GSH to reduce the molecules oxidized by ROS.

Other presently unknown genetic differences could account for the results shown here. There are examples in the literature of unusual redox responses in rodents and humans under conditions typically associated with oxidative stress. Different strains of female mice show an increase, a decrease, or no change in liver or kidney glutathione in response to HFD depending on the strain [34]. Likewise, when evaluating the effect of exercise on humans by quantifying markers of oxidative stress (F2-isoprostanes, protein carbonyls, and glutathione), most individuals exhibit increases in plasma F2-isoprostanes and protein carbonyls and decreases in glutathione, confirming the existence of oxidative stress, but around 10–13% of the individuals exhibit a decrease in those markers and an increase in glutathione, indicating that they produced reductive stress [35].

## 5. Conclusions

Obesity resulting from a high-fat diet (HFD) produced cardiac hypertrophy in the presence of reductive stress in cardiac tissue. This finding challenges the prevailing view that oxidative stress due to obesity drives hypertrophy. It emphasizes the idea that reductive stress can be equally detrimental as oxidative stress in contributing to hypertrophy. This highlights the importance of maintaining a balanced redox state for a healthy heart. Future experiments should focus on the identification of specific redox-sensitive proteins that play a role in the promotion of hypertrophy in obese animals.

## 6. Limitations of This Work

These results obtained in male mice may not represent the response of cardiac tissue in human obesity. They may not represent the effects of HFD in female mice due to hormonal influences and metabolic differences in female hearts. There are marked differences in the metabolomic profile between female and male mice, specifically in the content of the Krebs cycle and fatty acid intermediates, amino acids, vitamins, and various other metabolites. This suggests that the cardiac metabolism is different in males and females [36]. Furthermore, studies have demonstrated that mitochondria isolated from female liver or heart tissues generate lower levels of hydrogen peroxide than those derived from male tissues [37,38]. The reasons for these differences have not been fully investigated. Other aspects, such as mouse strain and feeding length period, could have influenced the results. Also, ROS generations are compartmentalized in the cell, and they occur in discrete domains, and our measurements were performed in total cardiac homogenates, and we lack assessments of redox behavior across distinct cardiac domains.

## Figures and Tables

**Table 1:** List of primers used for q-PCR.

Gene	Gen Bank Accession Number	Primer Sequences (5′-3′) Forward(F) Reverse (R)	Length (bp)	Annealing T (°C)	Fragment Size (bp)
*β-MHC*	NM_001425737.1	F: AAGCAGCAGTTGGATGAGCG	20	55	133
		R: CCTCGATGCGTGCCTGAAGC	20		
*RCAN1.4*	NM_019466.4	F: CCCGTGAAAAAGCAGAATGC	20	55	141
		R: TCCTTGTCATATGTTCTGAAGAGGG	25		
*BNP*	NM_008726.6	F: CATGGATCTCCTGAAGGTGC	20	55	188
		R: CCTTCAAGAGCTGTCTCTGG	20		
*Collagen Type 1*	NM_007742.4	F: CTGACGCATGGCCAAGAAGA	20	55	94
		R: AGCATACCTCGGGTTTCCAC	20		
*Collagen Type 3*	NM_009930.2	F: TCCCCTGGAATCTGTGAATC	20	55	63
		R: TGAGTCGAATTGGGGAGAAT	20		
*GR*	NM_010344.4	F: CAGTTCCTCACGAGAGCCAG	20	55	123
		R: TCTCCACAGCAATGTACCCG	20		
*GPX4*	NM_001037741.4	F: TTACGAATCCTGGCCTTCCC	20	55	82
		R: CGGCTGCAAACTCCTTGATT	20		
*SOD1*	NM_011434.2	F: CCAGTGCAGGACCTCATTTT	20	55	216
		R: CACCTTTGCCCAAGTCATCT	20		
*SOD2*	NM_013671.3	F: GGCCAAGGGAGATGTTACAA	20	55	216
		R: GAACCTTGGACTCCCACA	18		
*Catalase*	NM_009804.2	F: TGCAGCTCCGCAATCCTAC	19	55	113
		R: CTCCGGTGGTCAGGACATCA	20		

**Table 2:** Physical and biochemical parameters in HFD-fed mice.

	Control	HFD	*p*
n	34	34	
Physical Parameters			
Body Weight (g)	28.2 ± 2.5	43.5 ± 4.7	<0.001
Heart Weight (mg)	139 ± 15	153 ± 16	<0.001
Tibia Length (mm)	16.98 ± 0.30	16.97 ± 0.40	ns
HW/TL (mg/mm)	8.22 ± 0.88	9.02 ± 0.95	<0.001
n	11	12	
Biochemical Parameters			
Glucose (mg/dL)	156 ± 48	250 ± 20	<0.001
Insulin (mIU/L)	3.6 ± 1.9	35.9 ± 11.9	<0.001
Triglycerides (mg/dL)	68 ± 14	86 ± 9	<0.001
Cholesterol (mg/dL)	82 ± 12	164 ± 24	<0.001

Biochemical parameters were measured after 6 h of fasting. Values are mean ± SD of the number of animals indicated on top. ns: not significant.

**Table 3:** Cardiac dimensions and fractional shortening in HFD-fed mice.

	Control	HFD	*p*
n	8	8	
Heart rate (BPM)	712 ± 24	756 ± 31	ns
LVID diastole, mm	0.260 ± 0.020	0.311 ± 0.025	ns
LVID systole, mm	0.151 ± 0.017	0.150 ± 0.021	ns
FS%	42.80 ± 2.22	49.78 ± 4.90	ns

LVID: Left ventricular internal diameter; FS: fractional shortening; HFD: high-fat diet. Values are given as mean ± SD. ns: not significant.

## Data Availability

Data is contained within the article.

## Abbreviations

The following abbreviations are used in this manuscript:

βMHC	myosin heavy chain β-isoform
BNP	brain natriuretic peptide
GSH	reduced glutathione
GSSG	oxidized glutathione
HFD	high-fat diet
NADH/NAD^+^	nicotinamide adenine dinucleotide reduced/oxidized form
NADPH/NADP^+^	nicotinamide adenine dinucleotide phosphate reduced/oxidized form
NOX4	NADPH oxidase 4
RCAN 1.4	regulator of calcineurin 1 isoform 4
ROS	reactive oxygen species
TBARS	thiobarbituric acid reactive substances
